# Determination of in vivo biological activities of *Dodonaea viscosa* flowers against CCL_4_ toxicity in albino mice with bioactive compound detection

**DOI:** 10.1038/s41598-021-92638-6

**Published:** 2021-06-25

**Authors:** Zhao-Wei Tong, Hina Gul, Muhammad Awais, Salina Saddick, Falak Sher Khan, Muhammad Gulfraz, Umara Afzal, Khizar Nazir, M. Y. Malik, Sami Ullah Khan, M. Ijaz Khan

**Affiliations:** 1 Department of Infectious Diseases, Huzhou Central Hospital, Zhejiang, 313000 Huzhou People’s Republic of China; 2 University Institute of Biochemistry and Biotechnology (UIBB), PMAS UAAR Rawalpindi, Rawalpindi, Pakistan; 3 Department of Biochemistry and Molecular Biology, Faculty of Sciences, University of Sialkot, Sialkot, Pakistan; 4 Department of Biological Sciences, Faculty of Science, King Abdulaziz University, P.O. Box 80203, Jeddah, 21589 Kingdom of Saudi Arabia; 5 Department of Biotechnology, University of Sialkot, Sialkot, Pakistan; 6 Department of Chemistry, Rawalpindi Women University, Satellite Town, Rawalpindi, Pakistan; 7 Department of Biosciences, University of Wah, Wah Cantt, 47040 Pakistan; 8 Department of Mathematics, College of Sciences, King Khalid University, Abha, 61413 Kingdom of Saudi Arabia; 9 Department of Mathematics, COMSATS University Islamabad, Sahiwal, 57000 Pakistan; 10 Department of Mathematics and Statistics, Riphah International University, I-14, Islamabad, 44000 Pakistan

**Keywords:** Biochemistry, Biotechnology

## Abstract

*Dodonaea viscosa* L.Jacq. is an evergreen shrub and native to Asia, Africa, and Australia. It has been used as traditional medicine in different countries. The foremost objective of the current study was to discover the protective potential of *D. viscosa* flowers Methanol (DVM) and Chloroform (DVC) extracts against CCL_4_ induced toxicity in mice. This study was intended to identify phytochemicals through HPLC, GCMS, and FT-IR, as well as in vitro antioxidant and in vitro anti-tuberculosis activity. Our comprehensive findings indicate that *Dodonaea viscosa* is valuable and widespread herbal medicine through therapeutic potentials for curing various ailments. *Dodonaeaviscosa* flowersare found to have a protective effect against oxidative stress produced by CCL_4_ in the liver, kidney, and spleen. The intake of DV extracts restored the level of hepatic enzymes (ALP, AST ALT, and Direct bilirubin), hematological parameters (RBCs, WBCs, and Platelets), total protein, and liver antioxidant enzymes (SOD, GPx, and CAT) after a decline in levels by CCL_4_. Histopathological results discovered the defensive effect of 300 mg/kg of DVM extract against CCL_4_ induced damage, thus having an improved protective effect compared to DVC and control. As a result of metabolite screening, the total flavonoids and total phenolics were present in abundance. A phytochemical investigation by HPLC identified gallic acid, epicatechin, cumeric acid, flavonoids, while GCMS estimated oleic acid (Octadecenoic acid) (C_18_H_34_O_2_), Stearic acid (C_18_H_36_O_2_), Ricinoleic acid (C_18_H_34_O_3_), and Cedrol (C_15_H_26_O). DVM extract exhibited resistance against in vitro *Mycobacterium tuberculosis* strains. So this study proposed that the protective effect of DV against oxidative damage induced in the liver, kidney, and spleen can be correlated to the antioxidant compounds.

## Introduction

Oxidation–reduction reactions fuel different biological processes^1^ andproduce free radicals such as lipid peroxides that damagecell membrane and alters enzyme activity^2,3^ and induces liver injury, cirrhosis, inflammation, and necrosis^4^.

A foremost cause of toxicity is exposure toalcohol, carbon tetrachloride, x-rays, and many other radiations, which stimulate reactive oxygen species and make different parts of the body susceptible to injury, i.e., liver, kidney, spleen, and many others^5,6^ and facilitate damage to the biological molecule and oxidize most of them^7^. CCL_4_ has been used in a mouse model for chemically induced hepatotoxicity as a result of oxidative stress. Carbon tetrachloride produces several reactive oxygen species and generates liver injury by cytochrome P450. Synthetic drugs are inadequate and possible to have adverse effects around the world. So to detoxify the xenobiotics, drugs, and infections, natural products have been extensively used in the treatment of various diseases due to the presence of antioxidants^8,9^. There is a lot of research to uncover the potential of antioxidants to scavenge free radicals and fight against cellular damage, aging, and other chronic diseases^10,11^. Among antioxidants, phenolics are extensively distributed in herbal medicines, act as anti-inflammatory compounds, and protect the anti-oxidant defense system in the liver^12,13^. Multi-drug-resistant tuberculosis (MDRTB) is growing very fast in medical science andislooking for a short-term and effective treatment measure^14^. Many plant species have always shown potential in ancient times to treat medical disorders with promising therapeutic agents^15^. The knowledge about a single chemical component in medicinal plants plays a critical role from the method of extraction to understanding pharmacological assay and possible toxicity^16^.

The evergreen shrub*Dodonaea visocosa* (DV) belongs to the family Sapindaceae, which comprises species (2000) and genera (150). This plant is native to Asia, Africa, and Australia and islocally known as vilayati Mehndi in South East Asia^17^. The previous studies reported the presence of phytochemicals such as triterpenoids, flavonoids, saponins, tannins, and coumarins in DV^18–20^. DV has been widely used in traditional medicineto treat inflammation, stomach ulcers, and liver aches^21,22^. The crude extractof DV revealed antioxidant, anti-microbial, anti-diabetic, gastro-protective activities and also reported hepato-protective activity in mice^23–26^. There is no detailedstudy reported regarding protective effects of flowers of DV, so taking it into accountpresent study was conducted to validate the potential of therapeutic activity of DV against liver and other organ toxicity. The foremost objective of this study was to discover the protective potential of *D. viscosa* flowers Methanol (DVM) and Chloroform (DVC) extracts against CCL_4_ induced toxicity in mice. This study was intended to identify phytochemicals by using HPLC, GCMS, and FT-IR as well as antioxidantand anti-tuberculosis activities.

## Material and methods

### Sample collection and preparation

The collection of *Dodonaea Viscosa*flowers were carried out during March and April from Murree hills, District Rawalpindi, Punjab, Pakistanaccording to the guidelines of institution (Quaid i Azam University, Islamabad)and alsothrough the permission of local community*.* With the Help of the Department of Botany Quaid-I-Azam University Islamabad, Pakistan, the plant species was detailed identifiedby Dr. Mushtaq ahmad (Plant taxonomist, QAU) before further processing. Voucher specimen HG052 was submitted to the herbarium of Pakistan (ISL) for future records. Cleaned specimens were subjected to shade drying followed by grinding and sieving. Dried the plant sample in a heating oven (37◦C) to eradicate excess moisture for absolute drying, and the pulverized material was prepared for further examination. Five hundred grams of plant powder was dissolved in Methyl alcohol and Chloroform for 5 days, and filtration of extracts was done with Whatman filter paper. Both extracts were evaporated with the help of a rotary evaporator, and crude dried extracts were stored in air-fitted vials for more processing.

### In vivo study

#### Selection and purchase of animals

Fifty male albino mice (body weight 55.2 ± 2.5 g) were purchased from NIH (National Institute for health) Islamabad. The experimental study was approved by the National Veterinary Lab Islamabad ethical committee adhering to the institution's guidelinesand also in compliance with ARRIVE guidelines. All the protocols having animal study were in acquiescence with the guidelines of the ethics committee. The mice were housed under controlled conditions and had free access to mouse chow (Feed Mills, Islamabad) and water ad libitum. Animals were cautiously monitored and kept up in standard house conditions.

#### Acute oral toxicity study

An acute toxicity study was done to select suitable doses of plant extracts for animals, as earlier reported by^27^. The bodyweight of animals was recorded before and after thestudy. Plant extracts were orally given to mice at the dose of 100–300 mg/kg body weight. After the dosage, animals were meticulously observed after 24, 48, and 72 h for the development of any toxicological symptoms. Animals were euthanized on 21 days of the experiment.

#### Experimental design

Animals of the same age were divided randomly into 10 groups, and each group contains 5 animals.

**Table:** 

1	Normal control group	Normal feedwas given without any treatment for 21 days
2	Olive oil group	1 ml of olive oil was given orally with their feed up to 21 days
3	CCL_4_ group (− ve control)	CCL_4_ (1 cc/kg b. w) was induced by the intra-peritoneal wayandgiven normal feed
4	100 mg Methanol group	This group was induced CCL_4_ and after that100 mg/kg b. w of methanol extractswas orally given with normal feed
5	200 mg Methanol group	Animals were given CCL_4_ + 200 mg/kg b. w of methanol extracts with normal feed
6	300 mg Methanol group	Animals were provided CCL_4_ + 300 mg/kg b. w of methanolic extract
7	100 mg Chloroform group	This group was administered CCL_4_ + 100 mg/kg b. w of chloroform extract
8	200 mg Chloroform group	The group was nourished with 200 mg/kg b. w of chloroform extract after CCL_4_
9	300 mg Chloroform group	After CCL_4_, 300 mg/kg b. w (chloroform extract) was given
10	Silyamrin group	CCL_4_ + 100 mg/kg body weight of Silymarin (standard drug)

At 20 days of the experiment, mice were kept for fasting for 12 h and animals were anesthetized and euthanized with sodium pentobarbiton. After anesthesia (21 days) whole blood was obtained from the heart by cardiac puncture. To get the serum, place the blood sample tube to clot for 30 minand subjected to centrifugation (3000 rpm for 10 min). Animals were sacrificed through cervical dislocation, and organs were collected and then rinsed using ice-cold saline solution and kept at − 20 °C for further analysis. The weight of collected organs from all the groups was recorded. For biochemical analysis, phosphate buffer saline was used to the homogenized liver (one part), then centrifuged at 3000 rpm for 20 min and supernatant was stored at − 20 °C. For the histopathological study, the liver, kidney, and spleen were stored in formalin solution (10%).

#### Analysis of blood samples

The serum biomarkers alanine aminotransferase (ALT), aspartate aminotransferase (AST), alkaline phosphatase (ALP), and bilirubin were examined by using an auto-analyzer with AMS diagnostic kits (Italy). RBC (red blood cells), WBC (White blood cells), and platelets in blood samples were estimated with the method reported by^28^.

#### Antioxidant enzymes

Liver homogenates were used toevaluate oxidative defense markers (antioxidant enzymes). Catalase (CAT) and superoxide dismutase (SOD) activity was measured by using the protocol^29^, Glutathione peroxidase (GPx) was calculated by the method of^30^, and total protein was evaluated by the method suggested by^31^.

#### Histopathological study

Mice organs (liver, kidney, and spleen) were removed carefully after sacrifice and preserved in formalin (10%). Test specimen were fixed, dehydrated in alcohol, cleaned in xylene, and then inserted into molten paraffin wax. Paraffin sections were cut into 5 µm thickness by using a microtome, and obtain tissues were mounted on slides and deparaffinized. Tissue sections were processed to staining with Ehrlich’s hematoxylin and eosin counterstained (H&E) and examined under a light microscope^32,33^.

### Determination of total phenolic and total flavonoid content

The total phenolic content of extracts was measured by using Folin–Ciocalteu reagent^34^. Results were expressed as grams of gallic acid equivalents per 500 g/dry weight. The total flavonoid content of the extracts was measured using colorimetric assay^35^. Results were expressed by using grams of quercetin equivalents per 500 g/dry weight.

### Antioxidant activity

#### Chemicals required

Methyl alcohol, ethyl alcohol, chloroform, DPPH, ABTS, Hydrogen peroxide, EDTA, Formalin, Xylene, Heamotoxylin, Essen, KH_2_PO_4_ buffer, ALT Alanine aminotransferase, AST Aspartate aminotransferase, ALP alkaline phosphatase, and bilirubin were obtained. Analytical grade solvents and reagents were purchased from local dealers of Sigma Aldrich and Merck.

#### DPPH assay

This process is used to calculate the scavenging capacity of the sample by the protocol with some modifications^36^. 2 ml aliquot of DPPH (2,2-diphenyl-1-picrylhydrazyl) was poured into each concentration of plant sample ranged from 20 to 100 µg/ml. This mixture was incubated at 37 °C for 30 min in darkness. Standard or Positive controls were Ascorbic and Gallic acid. DPPH solution was taken as a negative control. Reading was taken recorded at 517 nm, and results were expressed in ascorbic acid equivalent AAE and gallic acid equivalent GAE. The experiment was done in a triplicate manner, and the inhibition percentage is obtained by the following formula

$${\text{DPPH}}\,{\text{\% }} = \left[ {{\text{A}}^{{\text{a}}} {-}{\text{A}}^{{\text{h}}} /{\text{A}}^{{\text{a}}} } \right] \times 100$$

A^a^—absorbance of reaction mixture exceptfor plant extract. A^h^—absorbance of a reaction mixture comprising plant extract. IC 50 (µg /mg) was measured by plotting scavenging percentage against extract concentration.

#### Iron chelating assay

The Iron Chelating method was described by^37^. Antioxidant potential of plant extract was assessed through incubation of reaction mixture that comprises ofdifferentconcentrations of plant sample extracts (20–100 µg/ml), 2 mM ferrous sulfate (1 ml), and 0.25 mM Ferrozine (1 ml). After stirring, let the mixture stand for10 min, and absorbance was read at 517 nm.

$${\text{Chelating}}\,{\text{rate}}\,{\text{\% }} = \left[ {{\text{A}}^{{\text{a}}} {-}{\text{A}}^{{\text{h}}} /{\text{A}}^{{\text{a}}} } \right] \times 100$$

A^a^—absorbance of control lacking plant extract. A^h^—absorbance of mixture with plant extract. Standard solutions or Positive controls (Ascorbic and Gallic acid) were used to make the calibration curve. IC_50_ was stated as µg AAE/mg and GAE/mg.

#### Hydroxyl radical scavengingassay

Plants extract concentrations 20 to100 µg/ml were investigated by adding 0.2 M Sodium phosphate buffer (7 pH), 2deoxyribose (10 mM), FeSO_4_-EDTA (10 mM), H_2_O_2_ (10 mM) and 525 µl of H_2_O. Put all the mixture into TCA (2.8%) and TBA (1%) and incubate at 90 °C for color development. Spectrophotometric reading was observed at 520 nm. Standard drugs (Ascorbic and Gallic acids) were taken as Positive control, and results were measured in AAE and GAE µg/mg^38^.

$${\text{Scavenging}}\,{\text{activity}} = \left[ {1 - {\text{A}}^{{\text{h}}} /{\text{A}}^{{\text{a}}} } \right] \times 100$$

where A^a^—absorbance of the mixture (without plant sample extracts) and A^h^—absorbance of a mixture containing plant sample.

#### ABTS (2,2-azinobis [3-ethylbenzothiazoline-6-sulfonate]) radical cation decolorization assay

Plant extracts were analyzed through the enhanced ABTS + radical cation scavenging capacity by some modification^39,40^. ABTS + mixture was prepared by adding 3 mM ABTS (2,2-azinobis [3-ethylbenzothiazoline-6-sulfonate]) and potassium persulfate (2.5 mM). Leave the solution in the dark for 12 h. To measure ABTS + activity, ABTS + solution (3 ml) was taken with different concentrations of plant extract (20 to100 µg/ml). Optical density was measured at 734 nm. Standard drug used was Ascorbic and Gallic acids.

$${\text{Percent}}\,{\text{Scavenging}}\,{\text{potential}} = \left[ {{\text{A}}^{{\text{a}}} {-}{\text{A}}^{{\text{h}}} /{\text{A}}^{{\text{a}}} } \right] \times 100$$

A^a^—absorbance of control; A^h^—absorbance of plant extract.

#### Reducing power assay

FRAP (Ferric ion reducing power) was determined by the method that involved the mixing of each plant sample concentrations (20–100) µg/ml) with phosphate buffer (0.2 M) and potassium ferricyanide (0.1%). Allow the mixture to incubate in a water bath for 20 min. Subsequently, add trichloroacetic acid (10%), and the mixture was centrifuged at 3000 rpm for 10 min. The supernatant was mixed in distilled water (2 ml) andferric chloride (0.01%) and set it down for incubation. Blank and samples were interpreted at 700 nm. Standard compounds, i.e., Ascorbic and Gallic acid, were utilized as a positive control. Results were quantified as AAE and GAE (µg/mg)^41,42^.

#### Hydrogen peroxide scavenging activity (H_2_O_2_)

Hydrogen peroxide scavenging activity was described according to the protocol of^43^. Reaction mixture comprised of H_2_O_2_ solution 4 Mm (prepared in phosphate buffer) with different plant concentrations (20–100) µg/ml) followed by incubation for 10 min at room temperature. The reading was observed at 230 nm against a blank solution comprising phosphate buffer with 7.4 pH. Gallic acid and Ascorbic acid were used as standard and expressed in GAE and AAE (µg/mg).

$${\text{Scavenging}}\,{\text{activity}}\,{\text{\% }} = \left[ {{\text{A}}^{{\text{a}}} {-}{\text{A}}^{{\text{h}}} /{\text{A}}^{{\text{a}}} } \right] \times 100$$

A^a^ absorbance of H_2_O_2_ and A^h^ is the absorbance of mixture with plant extract.

#### Superoxide assay

The activity was determined by NBT reduction as per the method of Beauchamp and Fridovich, 1971^44^. PMS (phenazine methosulfate) and NADH (nicotinamide adenine dinucleotide) systems produce superoxide radicals that condense nitro blue tetrazolium (NBT) to purple formazan. Add up 50 mM Phosphate buffer, 0.73 mM NADH, 20 mM PMS and 0.5 Mm NBT in numerous concentrations of sample (20 µg/ml) and incubate them for 20 min. Optical density was documented at 560 nm against blank to determine generated formazan. The positive control was Ascorbic acid and Gallic acid. Inhibition concentration was obtained from the formula:

$${\text{Scavenging}}\,{\text{percentage}} = \left[ {1 - {\text{A}}^{{\text{h}}} /{\text{A}}^{{\text{a}}} } \right] \times 100$$

where A^a^—absorbance except plant concentrate and A^h^—absorbance of mixture with plant distillate.

### Analysis of plant with high-performance liquid chromatography (HPLC)

Crude extracts analysis was performed using a Shimadzu HPLC (high-performance liquid chromatography) system, Tokyo, Japan, equipped with a C18 column (250 mm × 4.5 mm, 5 m) used for the separation at the flow rate of 1 ml/min. The column temperature was sustained at 40◦C followed by gradient pump and UV/Visible detector. HPLC grade methanol was used for extraction of the crude plant to prepare the tested sample. Before injection, the filtration of samples was done using a 0.2 μm PTFE filter, and the injection volume was 10 µl. The compounds were eluted usinga gradient elution of mobile phases A and B (Acetonitrile and 0.1% phosphoric acid; 36:64). Separation steps are as follows: 0 min-5% B, 15 min-15% B, 15 min-45% B, 5 min-90%B, and Conditioning cycle for 5 min along with the analysis of the following initial conditions. The UV–Vis detection was recorded at 280–285 nm at a current rate of 1 ml/min per 20 min retention time. Quercetin was used as standard, and all data were done in triplicates.

### GC–MS

#### Gas chromatography and mass spectrometry analysis

GC–MS system QP2010 model (Shimadzu®) equipped with Mass selective Detector and split–a split-less system of injection. The instrument was fitted with capillary column RTx-5MS (cross bond 5% diphenyl—95% dimethylpolysiloxane) with 30 m × 0.25 mm with 0.25 μm film thickness. At the rate of 1.2 ml/min, helium was being used as carrier gas. The temperature program of the column was started at 150 °C (1 min) then programmed at 4 °C/min to 150 °C (10 min). The temperature of the injector was 275 °C while the detector was at 250 °C. 0.2 µl volume was injected in split mode. A split ratio was 1:50, and the mass spectra were operated electron ionization at 70 eV in Selected Ion Monitoring (SIM) mode were maintained. The run time of the machine was 40 min. The relative percentage of the plant extract compounds was expressed in percentage with normalization of peak area.

#### Compounds identification

GC mass spectrum interpretation was conducted by employing the database of the National Institute Standard and Technology (NIST). The compound name, molecular weight, and structure of the test materials were determined. The percentage (%) of each compound was calculated by comparison of the average area to total area. The spectrum of unknown constituents was compared with the version 2005, software, and Turbo mass 5.2. The aim was to discover the individual compound or group of compounds that might show its current commercial and traditional roles^45,46^.

### Fourier transform infrared spectrophotometer analysis

The plant extract was analyzed for infrared spectrum analysis by FT-IR (Fourier transform Infrared) spectroscopy Shimadzu machine, IR affinity 1, Japan. At first, loaded samples were grounded by KBr (1:100 w/w) with scan range (400–4000 cm) and 4 cm^−1^ resolution. Samples components were subjected to structural characterization and indicated functional groups with chemical bond types^47^.

### Anti-tuberculosis activity

The anti-mycobacterial activity of *Dodonaea viscosa* L. was measured using the REMA method^48^. *Mycobacterium tuberculosis* strains bug 206 and bug 1972 and H37Rv were grown in 7H9 broth. Sample stock solutions were diluted using DMSO to get the final concentration ranging from 0.98 to 250 μg/mL. Rifampicin was used as positive control drug ranges 0.004 to 1 μg/mL. Add the bacterial culture (5 × 10^5^ CFU/mL) to each well of the 96-well plate and incubate at 37 °C. Viability was tested using resazurin, and the color change and fluorescence were examined in plates by using SPECTRAfluor Plus microfluorimeter (TECAN). Experiments were performed in triplicates. The lowest concentration resulting in 90% growth inhibition of M. tuberculosis. The MIC was defined as the lowest concentration results in the inhibition of 90% growth of M. tuberculosis.

### Statistical analysis

All the data were obtained in a triplicate manner, and results are presented as mean ± standard deviation. One-way ANOVA was used for the processing of results. Statistical analysis (Mean, standard deviation, probability, and Pearson coefficient correlation) was obtained via statistical software (Prism pad 7). The level of significance was considered at < 0.05.

## Results

### In vivo study

#### Acute toxicity and effect on weight

According to the results, acute toxicity manifested significant noticeable signs on the mice's body weight. The observed change was shown in Tables 1 and 2. In the present study, DV flowers with methanol and chloroform extracts found no devastating effect on mice. No mortality was found at the highest dose of 2000 mg/kg as it is considered the highest dose by OECD guidelines for any acute toxicity assay. Three plant concentrations 100 mg/kg, 200 mg/kg and 300 mg/kg were selected for the study.

**Table 1:** Protective effect of methanol extract of *Dodonaea Viscosa* flowers.

Treatments	Liver weight (g)	Kidney weight (g)	Spleen weight (g)	Rise (%) in body weight
Normal	5.60 ± 1.21	0.48 ± 0.1	2.080 ± 0.19	16 ± 2.9
Olive oil control	5.82 ± 0.94	0.51 ± 0.08	2.15 ± 0.2	20 ± 3.10
CCL_4_	3.96 ± 1.18	0.34 ± 0.12	3.34 ± 0.5	7.41 ± 2.05
Silymarin + CCL_4_	6.02 ± 0.749	0.5 ± 0.17	2.150 ± 0.31	22.27 ± 0.09
DV (100 mg/kg) + CCL_4_	4.016 ± 0.83	0.25 ± 0.045	3.11 ± 0.77	6.03 ± 0.67
DV (200 mg/kg) + CCL_4_	4.67 ± 0.71	0.33 ± 0.01	2.85 ± 0.3	10.060 ± 1.04
DV (300 mg/kg) + CCL_4_	5.105 ± 0.51	0.41 ± 0.1	2.49 ± 0.5	15.52 ± 1.75

Results were expressed in triplicate manner with mean ± SD.

**Table 2:** Protective effect of chloroform extract of *Dodonaea Viscosa* flowers.

Treatments	Liver weight (g)	Kidneyweight (g)	Spleen weight (g)	Rise (%) in body weight (g)
Normal	5.60 ± 1.21	0.48 ± 0.05	2.080 ± 0.1	16 ± 2.9
Olive oil control	5.82 ± 0.94	0.51 ± 0.02	2.15 ± 0.5	20 ± 3.10
CCL_4_	3.96 ± 1.18	0.34 ± 0.08	3.34 ± 0.2	7.41 ± 2.05
Silymarin + CCL_4_	6.02 ± 0.749	0.5 ± 0.1	2.150 ± 0.16	22.27 ± 0.09
DV (100 mg/kg) + CCL_4_	4.15 ± 0.92	0.24 ± 0.03	3.06 ± 0.8	7.22 ± 0.92
DV (200 mg/kg) + CCL_4_	4.62 ± 1.23	0.28 ± 0.14	2.76 ± 0.5	10.24 ± 2.31
DV (300 mg/kg) + CCL_4_	5.16 ± 0.92	0.36 ± 0.02	2.3 ± 0.33	12.94 ± 2.08

All the values are obtained in triplicate (mean ± standard deviation).

#### Liver enzymes

DV flower extracts were tested for protective effect on liver enzymes in albino mice. Group 1 and 2 showed normal enzymes level with no treatment. As a result of CCL_4_ treatment in (Group 3) slight to elevated changes were observed after 1, 2, 3, and 21 days in liver enzymes while compared with other groups. (Group 4–9) DVM showed a more restorative effect on liver enzymes than chloroform extract, and group 10 indicated hepatoprotective effect by Silymarineffect (Table 3).

**Table 3:** Estimation of liver enzymes from mice blood serum.

	ALT	AST	ALP	Direct bilirubin
Normal contro	38 ± 0.83	80.2 ± 4.1	110 ± 9.2	0.2 ± 0.001
Olive oil control	40 ± 5.2	63 ± 2	175 ± 20	0.32 ± 0.06
CCL_4_ control 1st day (24 h)	74.5 ± 7.7	90 ± 11	208 ± 5.1	1.0 ± 0.2
CCL_4_ control 2nd day (48 h)	85 ± 12	95 ± 3.5	225 ± 11	1.25 ± 0.04
CCL_4_ control 3rd day (72 h)	89 ± 14	109 ± 9.2	247 ± 2	1.7 ± 0.45
CCL_4_ control 21 day	120 ± 9	127.8 ± 8.6	294 ± 15	1.91 ± 0.08
Silymarin drug	66.4 ± 7.2	76.1 ± 12	185 ± 31	0.53 ± 0.09
DVM 100 mg + CCL_4_	62.5 ± 8	104 ± 20	264.8 ± 11	0.7 ± 0.08
DVM 200 mg + CCL_4_	61.8 ± 4.5	88 ± 11	236 ± 14	0.6 ± 0.01
DVM 300 mg + CCL_4_	53 ± 2	74.5 ± 6	197 ± 9.8	0.56 ± 0.02
DVC 100 mg + CCL_4_	77 ± 5	126 ± 7.1	357 ± 12.4	1.1 ± 0.2
DVC 200 mg + CCL_4_	72 ± 3	104.3 ± 5	283.6 ± 3.2	0.98 ± 0.05
DVC 300 mg + CCL_4_	69 ± 6	96 ± 4.3	229 ± 11	0.81 ± 0.07

Results are taken in a triplicate way with mean ± SD. Level of significance at < 0.05.

#### Hematological parameters

Induction of CCL_4_ resulted in a decline in RBCs, WBCs and plateletslevel. DV methanol and chloroform extract significantly increase the level of parameters (Table 4). DV methanol depicted greater RBCs and WBCs values closer to the normal control with significant results (p < 0.05).

**Table 4:** Hematological parameters of different groups of mice.

Groups	RBCs	WBCs	Platelets
Normal group	4.80 ± 0.06	5.02 ± 0.47	240 ± 6.9
Olive oil control	4.93 ± 0.2	5.50 ± 0.008	252 ± 10.1
CCL_4_ control 1st day (24 h)	3.05 ± 0.081	3.68 ± 0.03	170 ± 5.03
CCL_4_ control 2nd day (48 h)	2.73 ± 0.03	3.25 ± 0.15	155.2 ± 0.25
CCL_4_ control 3rd day (72 h)	2.40 ± 0.009	2.913 ± 0.092	140.01 ± 0.17
CCL_4_ control 21 day	1.88 ± 0.005	2.351 ± 0.11	100.59 ± 2.9
Silymarin drug	3.97 ± 0.12	4.802 ± 0.060	227.3 ± 4.12
DVM 100 mg + CCL_4_	3.7 ± 0.016	4.67 ± 0.04	240.3 ± 0.15
DVM 200 mg + CCL_4_	4.181 ± 0.004	5.002 ± 0.12	247.09 ± 0.21
DVM 300 mg + CCL_4_	4.62 ± 0.21	5.56 ± 0.01	256.6 ± 0.82
DVC 100 mg + CCL_4_	2.612 ± 0.093	3.205 ± 0.83	174.1 ± 0.26
DVC 200 mg + CCL_4_	3.05 ± 0.02	3.84 ± 0.131	198.3 ± 0.03
DVC 300 mg + CCL_4_	3.73 ± 0.1	4.291 ± 0.20	224.20 ± 0.19

#### Antioxidant enzymes

The results showed that the normal level of enzymes getsaltered due to CCL_4_ administration. After carbon tetrachloride induction, hightoxicity reduced the antioxidant enzymes. Silymarin drug (positive control) and plant extracts revealed positive results. Results obtained in a triplicate manner along with coefficient variation were < 0.05 (Table 5).

**Table 5:** Effects of *Dodonaea Viscosa* flowers on Antioxidant enzymes and total proteins.

Groups	CAT (m mol/min/mg protein)	SOD (U SOD/mg protein)	GPx (µmol/min/mg protein)	Protein tissue
Normal control	8.2 ± 2	10.15 ± 1.4	32.10 ± 3	3.15 ± 0.4
Olive oil	7.9 ± 0.5	10.23 ± 2	34.7 ± 4.5	3.2 ± 0.25
CCL_4_ 1st day (24 h)	5.94 ± 1.3	6.89 ± 0.7	22.40 ± 3.7	1.2 ± 0.11
CCL_4_ 2nd day (48 h)	5.28 ± 1.7	6.24 ± 1	20.47 ± 1.9	1.03 ± 0.2
CCL_4_ 3rd day (72 h)	5.03 ± 0.43	5.98 ± 1.4	18.16 ± 2	0.98 ± 0.3
CCL_4_ 21 day	4.75 ± 1.9	5.75 ± 0.6	17.83 ± 3	0.75 ± 0.08
DVM100 mg + CCL_4_	6.8 ± 0.2	7.79 ± 1	19.4 ± 1.2	1.58 ± 0.05
DVM200 mg + CCL_4_	7.3 ± 0.8	8.58 ± 2.3	23.0 ± 3.5	2.1 ± 0.4
DVM300 mg + CCL_4_	7.8 ± 1.03	10.7 ± 0.5	29.4 ± 2	2.8 ± 0.08
DVC100 mg + CCL_4_	5.9 ± 1.5	5.86 ± 1.09	20.1 ± 3	1.02 ± 0.5
DVC200 mg + CCL_4_	6.2 ± 2	6.9 ± 1.5	23.4 ± 0.7	1.71 ± 0.9
DVC300 mg + CCL_4_	6.57 ± 1	8.02 ± 1	25.8 ± 1	2.1 ± 0.71
Silymarin + CCL_4_	8.11 ± 1.2	11.62 ± 0.6	33.7 ± 1.5	3.22 ± 0.8

All the results were obtained in triplicate with mean and standard deviation.

#### Histopathology

**Figure 1:**
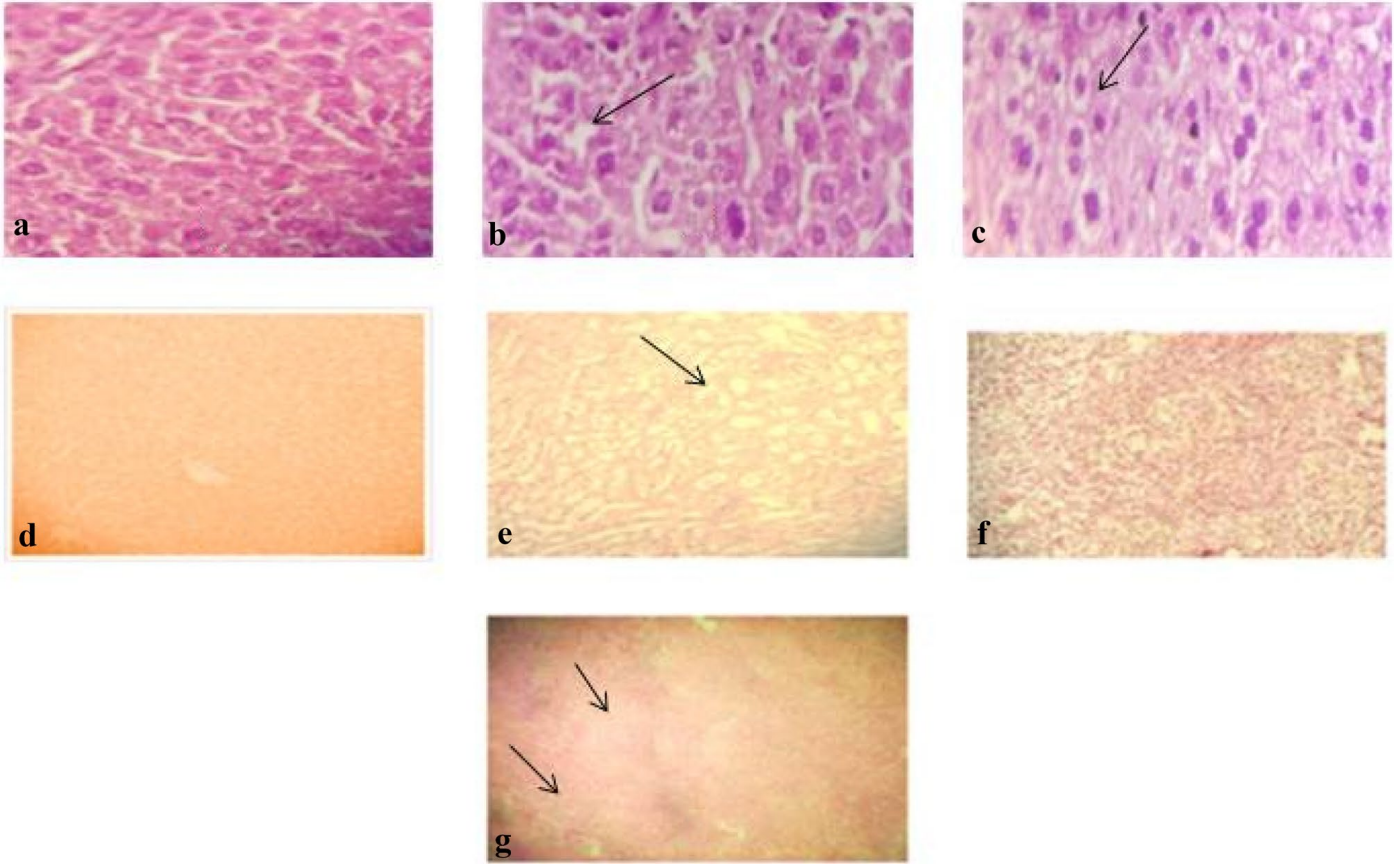
“**a**” showed the normal mice liver tissues, followed by “**b**” (arrow) showed the degeneration of hepatocytes. “**c**” presents recovery of degenerated hepatocytescaused by CCL_4_ induction. “**d**” CCL_4_ treated cellular structure of kidney. “**e**” DVM treated kidneyshowed slight recovery of renal tubules. “**f**” presents a normal spleen, while “**g**” arrow depicts normal lymphoid masses and red pulp in DVC-treated spleen.

Morphological changes in organ (Liver, kidney, and spleen) tissues were investigated by histopathological microscopy. CCL_4_ intoxication damaged the normal architecture of cells, and after 21 days of DV treatment, the cell structure of mice tissues was integrated. Several concentrations of plant extracts exhibited a protective effect on tissues by attenuating injuries, while methanol extract presented more supportive evidence on the cellular organization than DVC extract (Fig. 1).

The normal liver architecture depicts central vein, hepatic sinusoids as well as portal veins with normal appearance. Fibrosis and vascular irregularities, for instance, liver sinusoids alteration and central vein congestion, were seen in carbon tetrachloride mice. Renal histology revealed normal features like intact glomerular, tubular structure, bowman’s space and capillary tufts. Treated spleen showed white pulp containing normal lymphoid masses followed by extremely vascular red pulp, which was similar to normal mice histology.

### Phytochemical analysis and antioxidant assays

*Dodonaea viscosa* flower possessed total phenolic content 174 ± 4 mg/g dry weight and total flavonoid content 98 ± 7 mg/g, where the results are significantly different p < 0.05. DVM extract was evaluated against different antioxidant activities. The lowest IC_50_ was found in hydrogen peroxide assay 11.37 ± 0.4 mg/g dry weight and hydroxyl radical scavenging assay 19 ± 0.56 mg/g dry weight, which is greater than Ascorbic and Gallic acid. DVM also showed good activity to quench free radicals in DPPH free radical scavenging assay (Table 6).

**Table 6:** IC_50_ of different antioxidant assays.

	ABTS assay	Reducing power assay	DPPH assay	Iron chelating assay	Hydrogen peroxide assay	Hydroxyl scavenging Assay	Superoxide assay
*D. viscosa*	107.1 ± 11.4	75.59 ± 4	54.95 ± 2.1	20.7 ± 1.3	11.37 ± 0.4	19 ± 0.56	111.6 ± 2.1
Ascorbic acid	119 ± 7.9	25.7 ± 2	15.7 ± 3	29.2 ± .7	16.8 ± 2.1	24.5 ± 0.84	116.6 ± 2.8
Gallic acid	229 ± 15	39.2 ± 1	24.7 ± 2	34.8 ± 2	13.1 ± 1	26.2 ± 1	134.2 ± 5.6

The values were measured in µg/g.

### High-performance liquid chromatography

**Figure 2:**
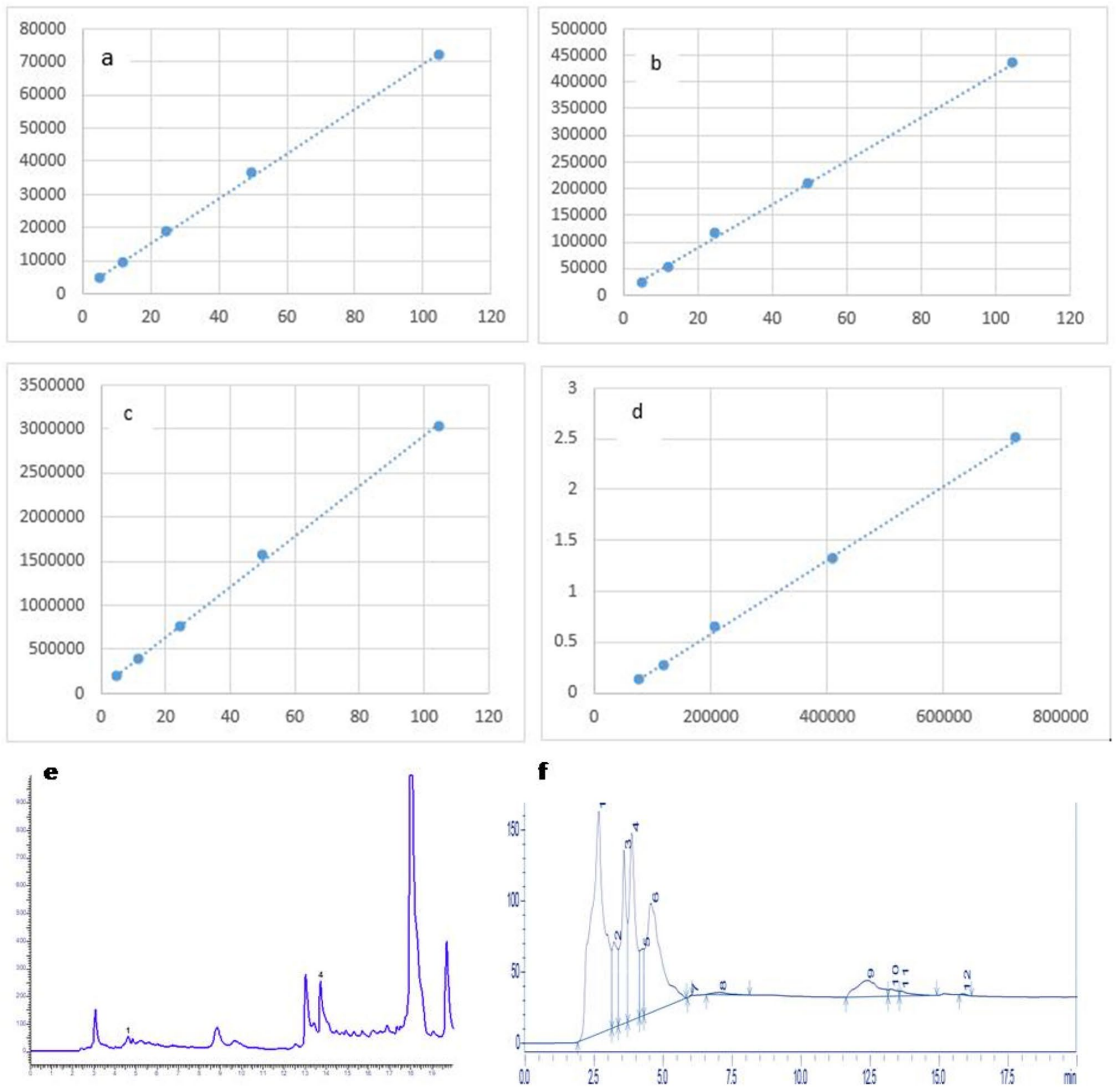
Different peaks on chromatograms showing different compounds. (**a**) Gallic acid standard graph (**b**) epicatechin standard graph (**c**) cumeric acid standard graph (**d**) quercetin standard graph (**e**) peaks showing gallic acid, epicatechin and cumeric acid from DVM (**f**) chromatogram peaks exhibited quercetin from DVM.

For the detection of some important medicinal compounds, HPLC analysis was carried out. Identification of peaks wasmade by comparing the retention time of the *Dodonaea viscosa* flower with standard compounds. The resulting peaks correspond to each compound were proportioned (Fig. 2). HPLC quantification of DVM flower extract identified the presence of gallic acid, epicatechin, cumeric acid, and quercetin compound. Whereas catechin has not been quantified in DV extract (Table 7). Gallic acid was the highest content observed with the quantity of 196.78 mg/kg.

**Table 7:** HPLC analysis of *Dodonaea viscosa* flower methanol extract.

Compounds	Area	Quantity (mg/kg)
Gallic acid	690,219	196.78
Catechin	0	− 1.04
Epichatechin	101,583	140.76
Cumeric acid	3,374,640	110.85
Quercetin	1,760,828	59.49

### Gas chromatography-mass spectroscopy (GC–MS) analysis

**Figure 3:**
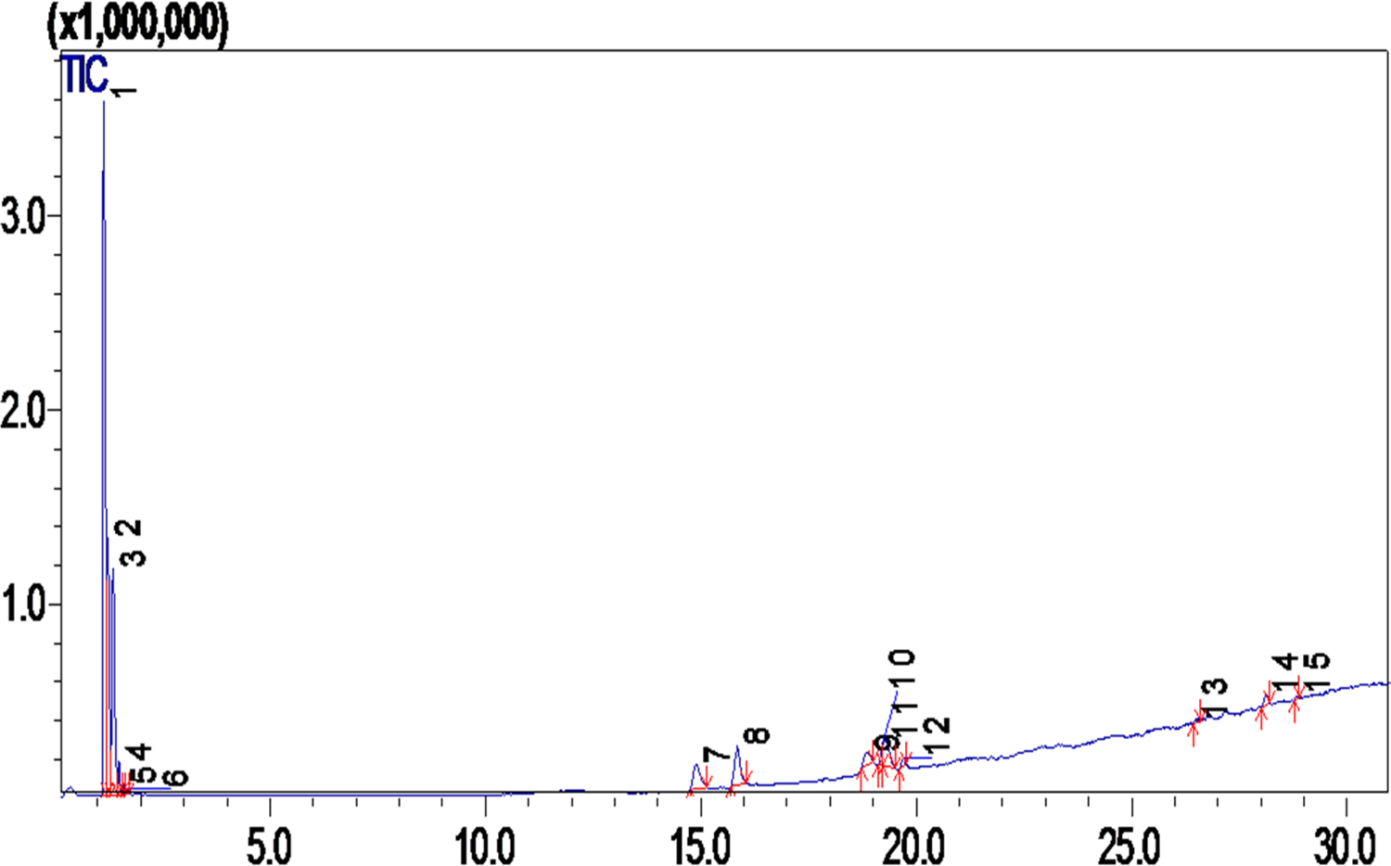
GCMS Chromatogram of *Dodonaea viscosa* flowers.

DVM extract is composed of volatile-based organic compounds, mainly fatty acids. Numerous compounds were identified by GC–MS, and the compound list followed by the corresponding GC–MS spectrum was presented in Table 8 (Fig. 3). Among all compounds, the most significant were Ascorbic acid (C_38_H_68_O_8_), Octadecenoic acid (C_18_H_34_O_2_), Ricinoleic acid (C_18_H_34_O_3_), Carboxylic acid (C_23_H_32_O_4_), Stearic acid (C_18_H_36_O_2_), and Cedrol (C_15_H_26_O).

**Table 8:** GCMS of *Dodonaea viscosa* flowers compounds.

Peaks	Compounds name	Formula	Molecular weight (amu)	Area %	Retention time (min)
1	Ethyl fluoride	C_2_H_5_F	48	44.66	1.132
2	Isobutyl alcohol	C_4_H_10_O	74	16.36	1.218
3	Isopentyl alcohol	C_5_H_12_O	88	16.78	1.350
4	Furanone	C_5_H_8_O_2_	100	1.58	1.488
5	Dimethyl Sulfoxonium formylmethylide	C_4_H_8_O_2_S	120	0.34	1.612
6	Isopentyl alcohol	C_7_H_14_O_2_	130	0.30	1.660
7	Ascorbic acid	C_38_H_68_O_8_	652	4.56	14.900
8	Ascorbic acid	C_38_H_68_O_8_	652	5.56	15.846
9	Octadecenoic acid	C_18_H_34_O_2_	282	2.11	18.858
10	Ricinoleic acid	C_18_H_34_O_3_	298	1.02	19.205
11	Octadecenoic acid	C_18_H_34_O_2_	282	4.30	19.278
12	Stearic acid	C_18_H_36_O_2_	284	0.74	19.697
13	Carboxylic acid	C_23_H_32_O_4_	372	0.40	26.460
14	Cyclopentanone	C_15_H_20_O	216	1.08	28.112
15	Cedrol	C_15_H_26_O	222	0.21	28.862

Data were obtained by triplicate readings with mean and standard deviation.

### Fourier transform infrared spectroscopy

**Figure 4:**
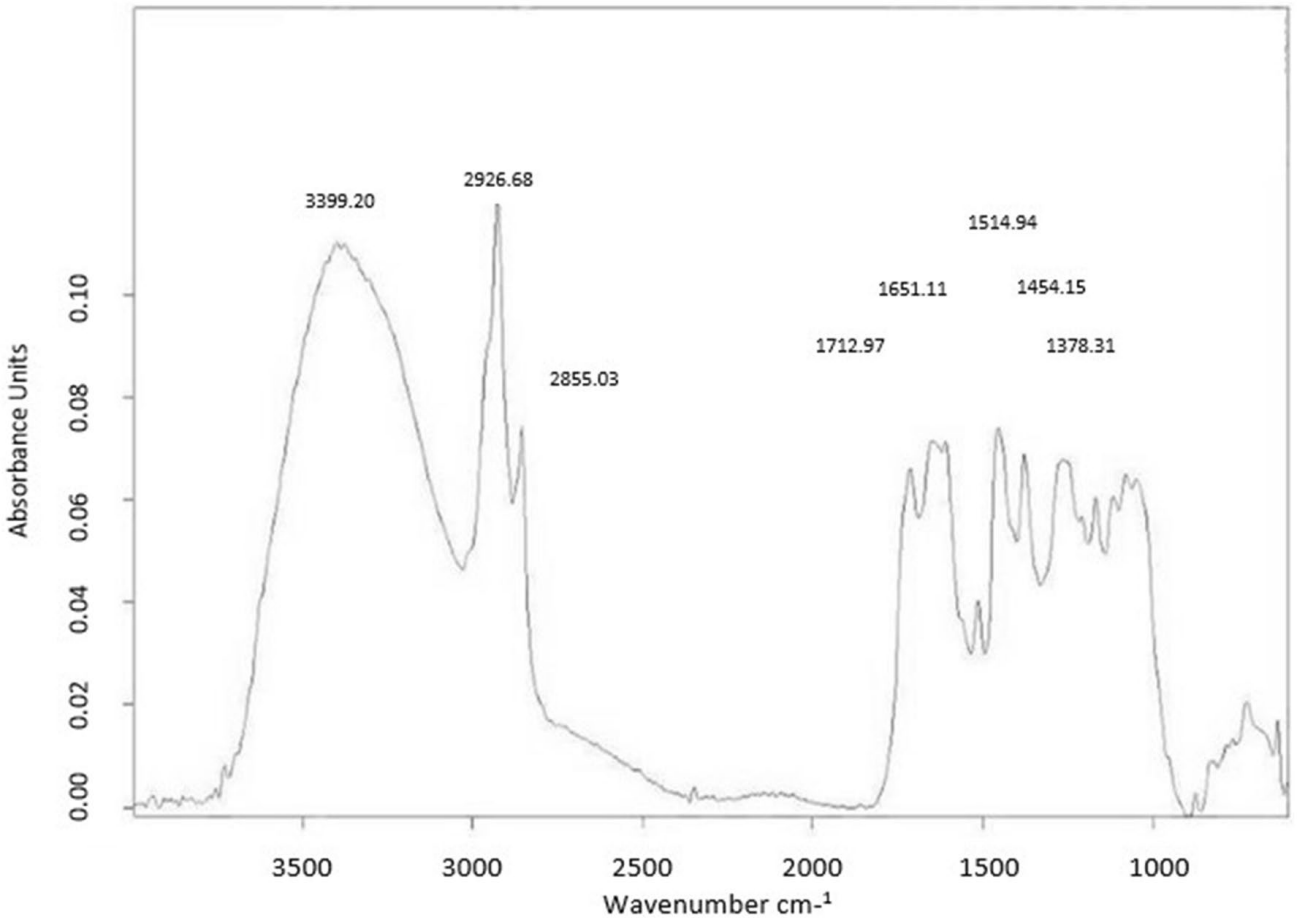
FT-IR analysis of *Dodonaea viscosa* flowers methanolic extract.

The most notable peaks in DVM were observed between 3500 and 2800 nm. A peak at 2926.68 nm belongs to O–H stretch, Carboxylic acids and then at 3399.20 nm correspond to O–H stretch, H-bonded which signifies Alcohols, phenols. Peaks ranging from 2900 to 700 nm belong to C–H stretch, C=O stretch, C–N stretch, and –C=C– stretch (Table 9; Fig. 4).

**Table 9:** FT-IR analysis of flowers of *Dodonaea Viscosa.*

Peaks	Wavelength	Bond	Functional group
1	3399.20	O–H stretch, H-bonded	Alcohols, phenols
2	2926.68	O–H stretch	Carboxylic acids
3	2855.03	C–H stretch	Alkanes
4	1712.97	C=O stretch	Carbonyl (general)
5	1651.11	–C=C– stretch	Alkenes
6	1514.15	N–O asymmetric stretch	Nitro compounds
7	1454.94	C–H Bend	Alkanes
9	1265.80	C–H wag (–CH_2_X)	Alkyl halides
10	1168.38	C–N stretch	Aliphatic amines
11	1079.18	C–N stretch	Aliphatic amines
12	724.89	C–H rock	Alkanes
13	632.98	C–Br stretch	Alkyl halides

### Anti-tuberculosis assay of *Dodonaea Viscosa* flowers

Anti-tuberculosis assay of DVM extract was screened out against *Mycobacterium tuberculosis* 3 strains bg 1972, bg 206 and H37Rv. 5 mg, 10 mg, and 50 mg/ml concentrations were used, and tuberculosis % inhibition was increased with the rise in concentrations. Plant extract showed resistance against all strains, but the highest activity was found against the H37Rv strain (Table 10). Minimum inhibitory concentration (MIC) was determined at 25 mg against H37Rv and bg 206. At the same time, standard drug (Rifampicin) exhibited MIC at 0.125 mg against H37Rv strain.

**Table 10:** Anti-tuberculosis activity of DV Methanol against different strains.

Isolates	Mean CFU on media			Percentage Inhibition		
	bg1972	H37Rv	Bg206	bg1972	H37Rv	Bg206
Control	130	140	150	130	140	150
5 mg/ml extracts	80	40	53	38	71	65
10 mg/ml	55	4	26	58	97	83
50 mg/ml	0	0	0	100	100	100

% Inhibition = Ccn − Ct/Ccn × 100, where Ccn = no of colonies in the control media slope, Ct = No of colonies in the Test media slope.

## Discussion

The present study demonstrated the in vitro and in vivo biological activities of *Dodonaea viscosa* flowers. The liver, kidney, and spleen are important parts of our body and are involved in different pivotal functions. The liver is one of the most important organs and played an important role in the detoxification of toxins^49,50^. CCl_4_ has been used to illuminate some mechanisms regarding various toxicities, i.e., lipid peroxidation, and cause necrosis, fibrosis, and apoptosis of cells^2^.

CCL_4_ is the toxin well known to produce chemical stimulated liver injurywhen it get metabolized into trichloromethyl radical (CCl_3_). This radical damages important cellular process by altering lipid metabolism and quantities of protein and then also induce mutations and produce hepatocellular carcinoma (HCC). Moreover, CCl_3_ oxygenation resulted in trichloromethylperoxy radicals (CCl_3_OO) thatlead to lipid peroxidation, polyunsaturated fatty acidsdestruction and lowered the permeability of the cellular membrane, and cause hepatic damage that is distinguished by fibrosis, cirrhosis, HCC, and Inflammation^51^.

Mice weight was reduced with the variation in weight of organs (Liver, Kidney, and Spleen). An increase in body weight owing to *Dodonaea viscosa* exhibited its protective effect. *Dodonaea viscosa* showed innocuous and protective to the mice, as reported earlier^52^. Level of liver enzymes ALT, ALP, AST, and direct bilirubin aimed to determine the sternness of damagedtissue^53^. Biochemical markers get altered by carbon tetrachloride and then restored by the treatment with plant extracts and standard drug Silymarin, indicating the usage of *Dodonaea viscosa* flowers against liver injury. CCL_4_ has been reported to increaseliver enzyme levels in some biochemical studies^54,55^. When there areunstablechangesobserved in the ALP level, it causes liver diseases^56^. Harmful changes in liver enzymes reflected several conditions likethe development of tissue necrosis, the decline in liver capacity (biosynthetic and catabolic), and alter normal structure of hepatocytes^57^.

Hematological parameters such as RBCs, WBCs, and platelets were also disturbed by CCL_4_ administration. Methanol and chloroform extracts of DV flower revealed positive effects on hematological parameters, which specify its suitability for managing blood cell disorders^58^. An endogenous enzyme CAT, SOD, and GPx involved in scavenging free radicals and declining normalenzyme levels indicate hepatic damage^58^. CCL_4_ reduces the level of antioxidant enzymes (CAT, SOD, and GPx) and total protein compared with the normal group and confirmsliver injury^59^, whereas the above factors were reinstated after administrating plant extracts. Intoxication of CCL_4_ in antioxidant enzymes can be improved using Medicinal plants^60,61^. Natural products have been investigated for the source of antioxidants that are being used for the hepato-protective activity. For the management of different diseases, flavonoids rich plants have shown protective effectsby decreasing serum markers with antioxidant and anti-inflammatory processes^62^.

Exposure to CCL_4_ leads to liver damage such as necrosis, fibrosis, and central vein alteration. In the kidney, it caused renal fibrosis, glomerular and tubular changes, while in the spleen, deterioration in white and red pulp occurred^63–65^. As a result of CCL_4_ toxicity, the cellular structure and function of the kidney rely onthe functional state of the liver^66^. Similar studies reveal that at high doses of extracts, liver, kidney, and spleen seemed nearly normal, with no observable gross morphological and histopathological modifications, supporting present findings^67^. Studies signify the use of *Dodonaea viscosa*against toxicity inan animal model and found to have revealeda protective effect for liver enzymes and attenuated the injury by diminishing the production of reactive oxygen species in hepatocytes^68^.

Methanol solvent was selected for further activities based onthe best results in the *in-vivo* study compared to chloroform. Preliminary screening of secondary metabolites resulted ina significant amount of total phenolic compounds and total flavonoid content. Phenolics and Flavonoids are considered singlet oxygen quenchers, radical scavengers, reducing agents, and hydrogen donors^69^. So, the analysis of the plant's total flavonoids and phenolic compounds is important to measure its antioxidant capacity. The results of the experiment presented strong antioxidant activities of *D*. *viscosa* flowers. The highest antioxidant activity of DVM was shown against hydrogen peroxide assay. DVM manifested great radical scavenging ability as follows Hydroxyl radical assay > iron chelating assay > DDPH assay > Reducing power assay > ABTS radical assay > Superoxide assay. In the current study, the reducing capacity of *D. viscosa* significantly decreases the complex of ferric cyanide to ferrous. The occurrence of antioxidants was determined by evaluating the ability of plant extract to form ferrous by reducing the ferric cyanide complex^70^. Reducing the power of plant compounds specifies its potential antioxidant capacity. High reducing power in a sample hasa great ability to donate the electron and free radicals and produce stable elements by accepting donated electrons, which terminates the free radical reaction^71^. Hydroxyl radicals are highly reactive free radicals in biological systems, and there are no specific enzymes present in humans to protect against them. Their presence in the human body causes oxidative DNA damage. Therefore, there is a need for a solution to scavenge ROS with natural products having scavenging activity. Due to the high reactivity of OH radicals, the antioxidant activity of scavenging hydroxyl radicals is important^72,73^. The most commonly used method for the evaluation of antioxidants is the DDPH assay. The quenching of DPPH measurement relies on the discoloration of the purple-colored 2,2-diphenyl-2-picryl-hydroxyl compound by antioxidant. Donor antioxidant decolorized DPPH radical by electron acceptance and measured quantitatively from variations in absorbance^74^. Furthermore, *D. viscosa* expressed significant radical scavenging activity against ABTS assay with a low value of IC_50._ All the essays are positive as well as significantly correlated with phenols and flavonoids.

HPLC quantified four compounds in DVM, i.e., gallic acid, epicatechin, quercetin, and cumeric acid. Quercetin is an iso flavonoid and flavonoid content (rutin and quercetin) was identified in the stem of *dodonaea viscosa*. The remedial aptitudes of *Dodonaea viscosa*are associated using pharmacological effects brought through the synergistic action of numerous constituents, i.e., flavonoids, saponins, di, and triterpenes, along with a combination of phenolics existing in the plant^75^. Flavonoids and diterpenoids are the richest secondary metabolites that werepreviously identified and isolated from Dodonaea^76^. These phenolic and flavonoid compounds revealed anticancer, antiallergic, antibacterial, antiviral, and anti-inflammatory activities^77^. The chemical compounds elucidated by GCMS were Oleic acid (Octadecenoic acid), Ascorbic acid, Ricinoleic acid, Stearic acid, Carboxylic acid, Cyclopentanone, and Cedrol. Fatty acids (Oleic, linoleic and linolenic acids) enriched food showed pleiotropic effects and used for the management of inflammation, hypertension, cardiovascular diseases, hyperlipidemia, reproductive ailments, immune system, and aggregation of platelets^78,79^. Research studies showed that Oleic acid exerts remedial effects on the human body, such as cancer, anti-inflammatory and autoimmune diseases, anda vital role in wound healing^80^. Ricinoleic acid is a significant unsaturated and hydroxylated fatty acid that depicts antipathogenic activity by deterring bacteria, viruses, mold, and yeast^81^. DVM showed very good activity against Tuberculosis strains. *Mycobacterium tuberculosis* is responsible for tuberculosis, which is among the fatal diseases. *DodonaeaViscosa* has been locally used in traditional medicines to treat tuberculosis^82,83^. Tested plant extract of DV flowers exhibited stronger resistance from all tested strains of *Mycobacterium tuberculosis* owing to the occurrence of bioactive components among the different concentrations of plant methanol extract that are probably anti-mycobacterial metabolites. Tuberculosis remains accountable for numerous mortalities around the world. During treatment, TB patients require extensive chemical analysis and eventually generate antagonistic effects on patient wellbeing. To diminish the use of unnatural resistant drugs, medicinally important plants contribute to great sureness as a potential reason for bioactive anti-mycobacterial metabolites^84^. A limited distinct species of genus Dodonaea have been extensively examined both chemically and pharmacologically. The most known species of genus Dodonaea is *D. viscosa* in literature^85^.

## Conclusion

*Dodonaea viscosa* is well-known plant species and widely possesses so many biological activities. Results showed the potential pharmacological effect of *Dodonaea Viscosa* against acute toxicity in albino mice, specifying its use against different diseases, most of all liver diseases. This plant showed significant biological activities such as antioxidant and anti-tuberculosis. The chemical composition of the plant is rich in antioxidant compounds, flavonoids, and phenols, and a rich source of Fatty acids, mainly oleic acid. These compounds could probably protect elevated hepatic enzymes caused by carbon tetrachloride and chronic tuberculosis. These curative effects are linked with the traditional use of this plant against different diseases. This plant might be used to extract promising drugs for the management of liver and multiple organs injury. The active compounds and their action mechanism, pharmacokinetics, toxicology, efficacy, and molecular mechanisms still need to be explored to attain integration into remedial practice.

## References

[1] Kong M, Chen XG, Xing K, Park HJ (2010). Anti-microbial properties of chitosan and mode of action: A state-of-the-art review. Int. J. Food Microbiol..

[2] Kandimalla R (2016). Bioactive guided fractions of *Annona reticulata* L. bark: Protection against liver toxicity and inflammation through inhibiting oxidative stress and proinflammatory cytokines. Front. Pharmacol..

[3] Kosecik M, Erel O, Sevinc E, Selek S (2005). Increased oxidative stress in children exposed to passive smoking. Int. J. Cardiol..

[4] Huang W, Metlakunta A, Dedousis N, Zhang P, Sipula I, Dube JJ, O’Doherty RM (2010). Depletion of liver Kupffer cells prevents the development of diet-induced hepatic steatosis and insulin resistance. Diabetes.

[5] Cesta MF (2006). Normal structure, function, and histology of the spleen. Toxicol. Pathol..

[6] Vedi M, Kalaiselvan S, Rasool M, Sabina EP (2013). Protective effects of blue green algae *Spirulina fusiformis* against galactosamine-induced hepatotoxicity in mice. Asian J. Pharm. Clin. Res..

[7] Liu CM, Ma JQ, Sun YZ (2012). Puerarin protects the rat liver against oxidative stress-mediated DNA damage and apoptosis induced by lead. Exp. Toxicol. Pathol..

[8] Sundararajan R, Haja NA, Venkatesan K, Mukherjee K, Saha BP, Bandyopadhyay A, Mukherjee PK (2006). Cytisus scoparius link-A natural antioxidant. BMC Complement. Altern. Med..

[9] Fu SY, Lau WY, Li AJ, Yang Y, Pan ZY, Sun YM, Wu MC (2010). Liver resection under total vascular exclusion with or without preceding Pringle manoeuvre. Br. J. Surg..

[10] Pareek A, Godavarthi A, Issarani R, Nagori BP (2013). Antioxidant and hepatoprotective activity of *Fagonia schweinfurthii* (Hadidi) Hadidi extract in carbon tetrachloride induced hepatotoxicity in HepG2 cell line and rats. J. Ethnopharmacol..

[11] Stevenson DE, Hurst RD (2007). Polyphenolic phytochemicals–just antioxidants or much more?. Cell. Mol. Life Sci..

[12] Nayak, B. S., Marshall, J. R., Isitor, G., & Adogwa, A. (2011). Hypoglycemic and hepatoprotective activity of fermented fruit juice of *Morinda citrifolia* (Noni) in diabetic rats. *Evidence Based Complement. Altern. Med.*, *2011*. 10.1155/2011/875293 PMC2958566 20981320

[13] Mojzer E, Knez Hrnčič M, Škerget M, Knez Ž, Bren U (2016). Polyphenols: Extraction methods, antioxidative action, bioavailability and anticarcinogenic effects. Molecules.

[14] Fauziyah PN, Sukandar EY, Ayuningtyas DK (2017). Combination effect of anti-tuberculosis drugs and ethanolic extract of selected medicinal plants against multi-drug resistant *Mycobacterium tuberculosis* isolates. Sci. Pharm..

[15] Sahu L, Jena S, Swain SS, Sahoo S, Chand PK (2013). Agrobacterium rhizogenes-mediated transformation of a multi-medicinal herb, *Boerhaavia diffusa* L.: optimization of the process and anti-microbial activity against bacterial pathogens causing urinary tract infections. Front. Life Sci..

[16] Wagner. H. In Handbook of Medicinal plants (Ed, Yaniv, Z. B., U.) *Haworth Press*, 2005.

[17] Anilreddy B (2009). Preparation, characterization and biological evaluation of some overview of *Dodonaea viscosa* Linn. J. Pharm. Sci. Technol..

[18] Perry, L. M., & Metzger, J. *Medicinal Plants of East and Southeast Asia: Attributed Properties and Uses*. MIT Press (1980).

[19] Pengelly, A. R. *Flavonoid Profile and Bioactivity of Dodonaea Viscosa (Australian Hop Bush)-an Indigenous Shrub*. University of Newcastle (2008).

[20] Getie M, Gebre-Mariam T, Rietz R, Höhne C, Huschka C, Schmidtke M, Neubert RHH (2003). Evaluation of the anti-microbial and anti-inflammatory activities of the medicinal plants *Dodonaea visco*sa, *Rumex nervosus* and *Rumex abyssinicus*. Fitoterapia.

[21] Veerapur, V. P., Prabhakar, K. R., Thippeswamy, B. S., Bansal, P., Srinivasan, K. K., & Unnikrishnan, M. K. (2010). Antidiabetic effect of *Dodonaea viscosa* (L). Lacq. aerial parts in high fructose-fed insulin resistant rats: a mechanism-based study. 21341538

[22] Rajamanickam V, Rajasekaran A, Anandarajagopal K, Sridharan D, Selvakumar K, Rathinaraj BS (2010). Anti-diarrheal activity of *Dodonaea viscosa* root extracts. Int J Pharm. Biol. Sci.

[23] Sachdev K, Kulshreshtha DK (1983). Flavonoids from *Dodonaea viscosa*. Phytochemistry.

[24] Ghisalberti EL (1998). Ethnopharmacology and phytochemistry of Dodonaea species. Fitoterapia.

[25] Ali H, Kabir N, Muhammad A, Shah MR, Musharraf SG, Iqbal N, Nadeem S (2014). Hautriwaic acid as one of the hepatoprotective constituent of Dodonaea viscose. Phytomedicine.

[26] Sastry KNS, Nayudamma Y (1966). Leucocyanidin from *Dodonaea viscosa* bark. Leather Sci..

[27] Wagner C, Ludwig L, Grotjahn MS, Khan Y (1987). Phytochemistry.

[28] Li M, He Y, Zhou Z, Ramirez T, Gao Y, Gao Y, Feng D (2017). MicroRNA-223 ameliorates alcoholic liver injury by inhibiting the IL-6–p47phox–oxidative stress pathway in neutrophils. Gut.

[29] Dacie JW, Lewis SM (1991). Practical Haematology.

[30] Misra HP, Fridovich I (1972). The role of superoxide anion in the autoxidation of epinephrine and a simple assay for superoxide dismutase. J. Biol. Chem..

[31] Flohé, L., & Günzler, W. A. (1984). Assays of glutathione peroxidase. In *Methods in Enzymology *(Vol. 105, pp. 114–120). Academic Press. 10.1016/s0076-6879(84)05015-1 6727659

[32] Lowry OH, Rosebrough NJ, Farr AL, Randall RJ (1951). Protein measurement with the Folin phenol reagent. J. Biol. Chem..

[33] Yakubu MT, Akanji MA, Oladiji AT (2007). Evaluation of antiandrogenic potentials of aqueous extract of *Chromolaena odoratum* (L.) KR leaves in male rats. Andrologia.

[34] Giribabu N, Karim K, Kilari EK, Salleh N (2017). Phyllanthus niruri leaves aqueous extract improves kidney functions, ameliorates kidney oxidative stress, inflammation, fibrosis and apoptosis and enhances kidney cell proliferation in adult male rats with diabetes mellitus. J. Ethnopharmacol..

[35] Sabir SM, Rocha JBT (2008). Antioxidant and hepatoprotective activity of aqueous extract of Solanum fastigiatum (false “Jurubeba”) against paracetamol-induced liver damage in mice. J. Ethnopharmacol..

[36] Moreno MIN, Isla MI, Sampietro AR, Vattuone MA (2000). Comparison of the free radical-scavenging activity of propolis from several regions of Argentina. J. Ethnopharmacol..

[37] Moon JK, Shibamoto T (2009). Antioxidant assays for plant and food components. J. Agric. Food Chem..

[38] Dinis TC, Madeira VM, Almeida LM (1994). Action of phenolic derivatives (acetaminophen, salicylate, and 5-aminosalicylate) as inhibitors of membrane lipid peroxidation and as peroxyl radical scavengers. Arch. Biochem. Biophys..

[39] Nagai T, Nagashima T, Suzuki N, Inoue R (2005). Antioxidant activity and angiotensin, I-converting enzyme inhibition by enzymatic hydrolysates from bee bread. Zeitschrift für Naturforschung C.

[40] Ashafa AOT, Grierson DS, Afolayan AK (2010). In vitro antioxidant activity of extracts from the leaves of *Felicia muricata* Thunb. an underutilized medicinal plant in the eastern cape province, South Africa. Afr. J. Tradit. Complement. Altern. Med..

[41] Dehghan G, Khoshkam Z (2012). Tin (II)–quercetin complex: Synthesis, spectral characterization and antioxidant activity. Food Chem..

[42] Hazra B, Biswas S, Mandal N (2008). Antioxidant and free radical scavenging activity of Spondias pinnata. BMC Complement. Altern. Med..

[43] Adedapo AA, Jimoh FO, Afolayan AJ, Masika PJ (2009). Antioxidant properties of the methanol extracts of the leaves and stems of *Celtis africana*. Rec. Nat. Prod..

[44] Aiyegoro OA, Okoh AI (2010). Preliminary phytochemical screening and in vitro antioxidant activities of the aqueous extract of *Helichrysum longifolium* DC. BMC Complement. Altern. Med..

[45] Beauchamp C, Fridovich I (1971). Superoxide dismutase: Improved assays and an assay applicable to acrylamide gels. Anal. Biochem..

[46] Daferera DJ, Ziogas BN, Polissiou MG (2000). GC–MS analysis of essential oils from some Greek aromatic plants and their fungitoxicity on *Penicillium digitatum*. J. Agric. Food Chem..

[47] Upadhyay RK (2015). GC-MS analysis and in vitro anti-microbial susceptibility of *Foeniculum vulgare* seed essential oil. Am. J. Plant Sci..

[48] Zargar M, Shameli K, Najafi GR, Farahani F (2014). Plant mediated green biosynthesis of silver nanoparticles using *Vitex negundo* L. extract. J. Ind. Eng. Chem..

[49] Palomino JC, Martin A, Camacho M, Guerra H, Swings J, Portaels F (2002). Resazurin microtiter assay plate: simple and inexpensive method for detection of drug resistance in *Mycobacterium tuberculosis*. Anti-microb. Agents Chemother..

[50] Gallagher EJ, LeRoith D, Stasinopoulos M, Zelenko Z, Shiloach J (2016). Polyol accumulation in muscle and liver in a mouse model of type 2 diabetes. J. Diabetes Comp..

[51] Scholten D, Trebicka J, Liedtke C, Weiskirchen R (2015). The carbon tetrachloride model in mice. Lab. Anim..

[52] Tu X, Zheng X, Li H, Cao Z, Chang H, Luan S, Zhang J (2015). MicroRNA-30 protects against carbon tetrachloride-induced liver fibrosis by attenuating transforming growth factor beta signaling in hepatic stellate cells. Toxicol. Sci..

[53] Zhang H, Yu CH, Jiang YP, Peng C, He K, Tang JY, Xin HL (2012). Protective effects of polydatin from *Polygonum cuspidatum* against carbon tetrachloride-induced liver injury in mice. PLoS ONE.

[54] Dong D, Zhang S, Yin L, Tang X, Xu Y, Han X, Peng J (2013). Protective effects of the total saponins from *Rosa laevigata* Michx fruit against carbon tetrachloride-induced acute liver injury in mice. Food Chem. Toxicol..

[55] Jose JK, Kuttan R (2000). Hepatoprotective activity of *Emblica officinalis* and Chyavanaprash. J. Ethnopharmacol..

[56] Nigatu TA, Afework M, Urga K, Ergete W, Makonnen E (2017). Toxicological investigation of acute and chronic treatment with *sGnidia stenophylla* Gilg root extract on some blood parameters and histopathology of spleen, liver and kidney in mice. BMC. Res. Notes.

[57] Middleton, E. (1998). Effect of plant flavonoids on immune and inflammatory cell function. In *Flavonoids in the Living System* (pp. 175–182). Springer. 10.1007/978-1-4615-5335-9_13 9781303

[58] Beji SR, Abidi A, Zemni R, Jameleddine BKS (2013). Antifibrosis effects of all-trans-retinoic acid in a rat model of bleomycininduced pulmonary fibrosis. Fundam. Clin. Pharmacol..

[59] Simeonova R, Kondeva-Burdina M, Vitcheva V, Krasteva I, Manov V, Mitcheva M (2014). Protective effects of the apigenin-O/C-diglucoside saponarin from *Gypsophila trichotoma* on carbone tetrachloride-induced hepatotoxicity in vitro/in vivo in rats. Phytomedicine.

[60] Gul H, Ahmad M, Zafar M, Sheeraz Ahmad M, Abid A, Hira S, Gulfraz M (2017). The in vitro and in vivo biological activities of the leaf of Cape Myrtle *Myrsine africana* L. Phytother. Res..

[61] Safhi MM (2018). Nephroprotective effect of Zingerone against CCl4-induced renal toxicity in *Swiss albino* mice: Molecular mechanism. Oxid. Med. Cell. Longevity.

[62] Sreelatha S, Padma P, Umadevi M (2009). Protective effects of *Coriandrum sativumextracts* on carbon tetrachloride-induced hepatotoxicity in rats. Food Chem. Toxicol..

[63] Ozturk F, Ucar M, Ozturk IC, Vardi N, Batcioglu K (2003). Carbon tetrachloride-induced nephrotoxicity and protective effect of betaine in Sprague–Dawley rats. Urology.

[64] Jiang JX, Chen X, Serizawa N, Szyndralewiez C, Page P, Schröder K, Török NJ (2012). Liver fibrosis and hepatocyte apoptosis are attenuated by GKT137831, a novel NOX4/NOX1 inhibitor in vivo. Free Radical Biol. Med..

[65] Seniutkin O, Furuya S, Luo YS, Cichocki JA, Fukushima H, Kato Y, Rusyn I (2018). Effects of pirfenidone in acute and sub-chronic liver fibrosis, and an initiation-promotion cancer model in the mouse. Toxicol. Appl. Pharmacol..

[66] Rincón AR, Covarrubias A, Pedraza-Chaverrí J, Poo JL, Armendáriz-Borunda J, Panduro A (1999). Differential effect of CCl4 on renal function in cirrhotic and non-cirrhotic rats. Exp. Toxicol. Pathol..

[67] Agbaje EO, Adeneye AA, Daramola AO (2009). Biochemical and toxicological studies of aqueous extract of *Syzigium aromaticum* (L.) Merr & Perry (Myrtaceae) in rodents. Afr. J. Tradit. Complement. Altern. Med..

[68] Arun M, Asha VV (2008). Gastroprotective effect of *Dodonaea viscosa* on various experimental ulcer models. J. Ethnopharmacol..

[69] Motamed SM, Naghibi F (2010). Antioxidant activity of some edible plants of the Turkmen Sahra region in northern Iran. Food Chem..

[70] Ahmed RG, Incerpi S, Ahmed F, Gaber A (2013). The developmental and physiological interactions between free radicals and antioxidant: Effect of environmental pollutants. J. Nat. Sci. Res..

[71] Kumari S, Elancheran R, Kotoky J, Devi R (2016). Rapid screening and identification of phenolic antioxidants in *Hydrocotyle sibthorpioides* Lam. by UPLC–ESI-MS/MS. Food Chem..

[72] Liu RH, Finley J (2005). Potential cell culture models for antioxidant research. J. Agric. Food Chem..

[73] Wang H, Gao XD, Zhou GC, Cai L, Yao WB (2008). In vitro and in vivo antioxidant activity of aqueous extract from *Choerospondias axillaris* fruit. Food Chem..

[74] Khan A, Anand V, Badrinarayanan V, Thirunethiran K, Natarajan P (2017). In vitro antioxidant and cytotoxicity analysis of leaves of *Ficus racemosa*. Free Radic. Antioxid..

[75] Wagner C, Fachinetto R, Dalla Corte CL, Brito VB, Severo D, Dias GDOC, Rocha JB (2006). Quercitrin, a glycoside form of quercetin, prevents lipid peroxidation in vitro. Brain Res..

[76] Simpson BS, Claudie DJ, Smith NM, Gerber JP, McKinnon RA, Semple SJ (2011). Flavonoids from the leaves and stems of *Dodonaea polyandra*: A Northern Kaanju medicinal plant. Phytochemistry.

[77] Maurya PM (2017). Phytochemical and pharmacological examination of *Achyranthes aspera* Linn. J. Pharmacogn. Phytochem..

[78] Eromosele CO, Eromosele IC (2002). Fatty acid compositions of seed oils of *Haematostaphis barteri* and *Ximenia americana*. Biores. Technol..

[79] Riediger ND, Othman RA, Suh M, Moghadasian MH (2009). A systemic review of the roles of n-3 fatty acids in health and disease. J. Am. Diet. Assoc..

[80] Campos SH, Reis de Souza P, Crema Peghini B, Santana da Silva J, Ribeiro Cardoso C (2013). An overview of the modulatory effects of oleic acid in health and disease. Mini. Rev. Med. Chem..

[81] Tunaru S, Althoff TF, Nüsing RM, Diener M, Offermanns S (2012). Castor oil induces laxation and uterus contraction via ricinoleic acid activating prostaglandin EP3 receptors. Proc. Natl. Acad. Sci..

[82] Asres K, Bucar F, Edelsbrunner S, Kartnig T, Höger G, Thiel W (2001). Investigations on anti-mycobacterial activity of some Ethiopian medicinal plants. Phytother. Res..

[83] McGaw LJ, Lall N, Meyer JJM, Eloff JN (2008). The potential of South African plants against Mycobacterium infections. J. Ethnopharmacol..

[84] Sankaranarayanan S, Bama P, Ramach J, Kalaichelvan PT, Deccaraman M, Vijayalakshimi M, Bama SS (2010). Ethnobotanical study of medicinal plants used by traditional users in Villupuram district of Tamil Nadu, India. J. Med. Plants Res..

[85] Gemechu A, Giday M, Worku A, Ameni G (2013). In vitro Anti-mycobacterial activity of selected medicinal plants against *Mycobacterium tuberculosis* and *Mycobacterium bovis* Strains. BMC Complement. Altern. Med..

